# An imaging review of extramedullary myeloma

**DOI:** 10.1186/1470-7330-15-S1-P12

**Published:** 2015-10-02

**Authors:** M Kay, A King, B Shepherd, E Rutherford, J Smart, K Tung

**Affiliations:** 1 University Hospital Southampton, Tremona Road, Southampton, Hampshire, SO16 6YD, UK

## Learning objectives

Numerous studies have demonstrated increasing overall survival in patients with multiple myeloma over the last 40 years. Novel agents including thalidomide and bortezomib used in induction therapy, together with autologous stem cell transplant in consolidation, have improved patient outcomes. Whilst extramedullary myeloma can be a presenting form of the disease, it is becoming increasingly recognised that extramedullary disease is common in patients who have relapsed disease, often after multiple lines of therapy. Whether this is due to either a possible “sanctuary site” effect from treatment in extramedullary tissues, prolonged overall survival leading to an evolution in the natural history of the disease, or the improved sensitivity of imaging techniques is not clear.

## Content organisation

We present a multimodality pictorial review of cases of extramedullary myeloma imaged at our tertiary oncology institution, demonstrating the common appearances on conventional imaging and the advantages of functional imaging such as PETCT in these cases.

## Conclusion

Being aware of the possibility of extramedullary disease, especially in cases of disease relapse, and the common imaging appearances, is vital due to the increasing incidence of this manifestation of the disease.

